# Disappearing Polymorphs Revisited

**DOI:** 10.1002/anie.201410356

**Published:** 2015-06-01

**Authors:** Dejan-Krešimir Bučar, Robert W Lancaster, Joel Bernstein

**Affiliations:** Department of Chemistry, University College London 20 Gordon Street, London WC1H 0AJ (United Kingdom); Faculty of Natural Sciences, New York University Abu Dhabi P.O. Box 129188, Abu Dhabi (United Arab Emirates); New York University Shanghai, Pudong New Area Shanghai 200122 (China); Department of Chemistry, Ben-Gurion University of the Negev Beer Sheva, 84120 (Israel)

**Keywords:** crystallization, drug formulation, nucleation, polymorphism, solid-state chemistry

## Abstract

Nearly twenty years ago, Dunitz and Bernstein described a selection of intriguing cases of polymorphs that disappear. The inability to obtain a crystal form that has previously been prepared is indeed a frustrating and potentially serious problem for solid-state scientists. This Review discusses recent occurrences and examples of disappearing polymorphs (as well as the emergence of elusive crystal forms) to demonstrate the enduring relevance of this troublesome, but always captivating, phenomenon in solid-state research. A number of these instances have been central issues in patent litigations. This Review, therefore, also highlights the complex relationship between crystal chemistry and the law.

## 1. Introduction

There is a continual and increasing demand for crystalline molecular materials with specific, fit-for-purpose physicochemical properties.^1^–^6^ Interest in polymorphism, crystallization, and (in industry) in robust process development has surged over the last two decades,^7^, ^8^ as evidenced by the immense growth in knowledge concerning the design, preparation, and characterization of crystalline materials.^9^ This expanding interest and demand for promising materials drives investigations of the solid form (i.e. polymorphs, solvates, hydrates, and amorphous materials) landscapes^8^, ^10^ of potentially relevant compounds, with the goal of identifying the optimally performing solid among them.

A broad range of crystallization techniques is generally employed to search for the most stable crystal form in hundreds or (in some cases) thousands of experimental attempts.^11^ New crystal forms can, however, emerge unexpectedly long after the carefully designed and executed screening experiments are completed. Such a sudden emergence of a new crystal form can be unsettling and problematic, especially in the late stages of a product development or even following launch, because the newly emerged form can exhibit different (possibly undesired) properties. Equally disruptive is the emergence of a thermodynamically more-stable crystal form, in accord with Ostwald’s Rule of Stages,^12^ concurrent with the disappearance of the less-stable known forms that signal a loss of control of the production process. While it may create roadblocks in the development process or even the marketed product of the solid form of a compound of interest, the consequences of the appearance of a new form are not necessarily negative. The serendipitous appearance of a new form may provide a substance with improved characteristics.

Unfortunately, our current understanding of the mechanisms and processes involved in the nucleation and growth of crystals is still insufficient for precise control over the formation or disappearance of a polymorph (or any other crystal form).^13^, ^14^ Nearly twenty years ago, Dunitz and Bernstein presented an overview of the disappearing polymorph phenomenon^15^ that has captivated and intrigued solid-state scientists since. In their review, Dunitz and Bernstein voiced their belief that crystal forms do not disappear permanently; on the contrary, once a solid form has been obtained, in principle it can always be reproduced if the right experimental conditions are met.^15^–^18^ In the same spirit as the earlier survey, this Review aims to discuss selected recent occurrences of disappearing polymorphs and of elusive crystal forms that have not only triggered the curiosity of researchers, but have also affected the business of pharmaceutical and health care companies. These examples illustrate how apparently stable polymorphs can suddenly disappear, and how elusive crystal forms can be prepared given the availability of conditions specifically designed to promote their formation. The uncontrolled loss of a crystal form can have serious consequences, and there is thus an urgent need to develop methods that provide absolute control over crystal nucleation and growth,^13^, ^14^ which is still an art, rather than a routine procedure.^19^

In addition to citing examples of disappearing polymorphs from the literature and our own laboratories, the 1995 review dealt with a number of issues that are still the subjects of debate. There have also been a number of patent litigations in which the same issues have arisen and have been interpreted variously by the courts. We will deal initially with those aspects of the subject and follow with the descriptions of a number of recent cases of disappearing polymorphs (and other crystal forms), as well as further details on some of those previously cited.

## 2. Disappearing Polymorphs—The Concept and Misconceptions

One of us (J.B.) recently recounted the genesis of the 1995 review,^20^ which was based on earlier cases in the laboratories of both Bernstein and Dunitz as well as additional examples we had encountered in the course of our involvement in the ranitidine hydrochloride litigations. In the twenty-year interim we have experienced numerous additional examples in which the phenomena described therein were either misinterpreted or misunderstood. Hence, we review some of those here.

### 2.1. The Concept

As we described in the section of the 1995 review headed “Vanishing Polymorphs”, a disappearing polymorph refers to a crystal form that has been prepared at least once and whose existence has been established experimentally by some observation or measurement. Subsequent attempts to prepare the same crystal form by the same procedure lead to a different crystal form, alone or together with the old one. If a mixture appears in the first instance, then very often in subsequent preparations the new form dominates and the old form is no longer obtained.

The phase rule limits to one the number of stable crystal forms that may exist under a specific set of conditions. The old—“disappeared”—form is generally less stable than the new one under those specific conditions. In thermodynamic terms, it is metastable, although that does not necessarily imply that it would spontaneously convert into a more stable form; it only means that it is at a higher energy minimum than the most stable state. To invoke a familiar example: diamond is metastable with respect to graphite; nevertheless, as is widely advertised, “diamonds are forever”.

The fact that a crystal form once existed, but is now difficult to prepare by the same method that was previously used, does not mean that it is impossible to prepare again. It has not been relegated to the “crystal form cemetery”.^21^ Every crystallization is a competition between kinetic and thermodynamic factors. As noted in the last sentence of the 1995 review, “*it is* always *possible to obtain [the old form] again; it is only a matter of finding the right experimental conditions*”—thermodynamic and kinetic.

Recovering a crystal form that has disappeared may require considerable time and effort and invoke some inventive and creative chemistry. The examples given below will demonstrate the kinds of strategies that have been employed to recover crystal forms that have disappeared.

### 2.2. Seeds and Seeding

The 1995 review also contains a section headed “Seeding”. Intentional seeding is a well-known technique for inducing crystallization and is widely used, especially in the pharmaceutical industry. Unintentional seeding arises from the presence of small amounts—indeed, in principle one particle is sufficient—of the solid material that is present even as a contaminant. As we noted earlier, “*Unintentional seeding is often invoked as an explanation of phenomena which are otherwise difficult to interpret. We shall argue in favor of this explanation, although there is no consensus about the size and range of activity of such seeds, which have never actually been directly observed*.”^15^

The situation this statement describes has led to considerable controversy, particularly in the framework of patent litigations involving crystal forms. That controversy very much represents the clash between the cultures of science and the law, and in light of that controversy it seems appropriate, indeed compelling, to put the phenomenon of unintentional seeding into a proper scientific perspective in this Review.

Virtually every chemist has at some time attempted to crystallize a compound. Crystallization is perhaps the classic method of purification, and the technique is one of the first mentioned in purification methods in any undergraduate organic chemistry laboratory textbook. Practicing chemists soon learn, often simply by experience, that it is frequently very difficult to crystallize a newly synthesized substance, while subsequent crystallizations are considerably more facile. The situation was documented over half a century ago by Wiberg in his classic text “Laboratory Technique in Organic Chemistry” in the section entitled “Inducing Crystallization”: “*When a compound is prepared for the first time in a laboratory, it is often observed that it is relatively difficult to effect crystallization. However, once the compound has been obtained in the crystalline state, it is usually easy to effect crystallization, and it has been suggested that after initial crystallization crystal nuclei are present in the laboratory and induce crystallization*”.^22^ In the current context those nuclei are unintentional seeds.

Many laymen are initially skeptical about a phenomenon caused by particles that cannot be seen, although very few would accept an invitation for a casual—and unprotected—visit to the pneumonia ward at their local hospital. The approximate limit of visual detection for the naked eye is a crystal that weighs approximately 10^−6^ g. We pointed out earlier that a speck of that size contains approximately 10^16^ molecules and while there are various estimates of the size of a critical nucleus that could act as a seed even the largest—a few million molecules^23^—would mean that an invisible particle could contain up to 10^10^ of such unintentional seeds.

Where do these microscopic particles come from? As noted elsewhere, depending on our location, the air contains a vast number of submicroscopic particles. For a normal urban environment there are approximately 10^6^ airborne particles of 0.5 micrometer diameter or larger per cubic foot, the number being reduced by an order of magnitude in an uninhabited rural environment. A sitting individual generates roughly one million dust particles (≥0.3 micrometer diameter) per minute (a visible particle is usually ≥10 micrometers).^24^ Clean rooms for various purposes (e.g. surgery, biological or pharmaceutical preparations, semiconductor fabrication) employ very sophisticated technology to remove these particles and to prevent subsequent contamination. Therefore, the possible presence of seeds of a newly formed polymorph in a laboratory, a manufacturing facility, or any location having been exposed to that form cannot be casually dismissed; indeed its presence would be hard to avoid. In his comprehensive monograph on crystallization, Mullin notes that, “*Atmospheric dust frequently contains particles of the crystalline product itself, especially in industrial plants or in laboratories where quantities of the material have been handled……Once a certain crystalline form has been prepared in a laboratory or plant, the working atmosphere inevitably becomes contaminated with seeds of the particular material*.”^23^

So much for the atmosphere. What about the crystallizing medium, usually a solution? The normal determination that dissolution has been completed is made by visual inspection. If the solution is clear to the human eye all the solute is assumed to be in solution. Mullin has also pointed out that *“aqueous solutions as normally prepared in the laboratory may contain >10*^*6*^ *solid particles per cm^3^……”*.^23^ These can be impurities or particles of the solute that have not undergone complete dissolution, and can serve as seeds for the subsequent crystallization.

The presence and influence of microscopic seeds and their influence on crystallization is thus well established. Nevertheless, it is difficult for many who lack practical laboratory experience to accept their existence. In the history of chemistry there have been many instances of inductive reasoning in understanding chemical phenomena. The existence of atoms was proposed and accepted for nearly two hundred years before an atom was actually “seen”. Yet no chemist doubts the existence of atoms or the ability to make and break bonds between them.

The presence and influence of seeds may be invoked to explain the disappearance of one crystal form at the expense of a new form. In such a case, the unintentional seeding by the new form may be quite aggressive, preventing the crystallization of the old form. However, there is no intrinsic reason why every system is influenced by such aggressive unintentional seeding. There are many known examples of multicrystalline materials in which the various forms can be prepared and maintained in the known presence of other forms. As for polymorphism in general, **every system is unique** and must be individually studied and characterized to understand how to prepare and characterize each form.

### 2.3. “Universal Seeding”

The publicity surrounding some cases of aggressive unintentional seeding led to discussions, particularly in legal circles, of the alleged phenomenon of universal seeding—that is, in some cases of disappearing polymorphs, when the old form could not be made by the old process somehow, there was an implication that the entire universe must be seeded. To put the matter to rest it is important to quote a footnote from the 1995 review: “*The claim for ‘universal seeding’, taken literally, is obviously absurd. After all, the universe is estimated to contain about a millimole of stars, so one seed per star (per solar system)—not much—would need about 100 kg of the compound in question (M_r_≈100)*”.

A number of cases of aggressive seeding have attained considerable notoriety, and these will be described below. In instances where various locations at considerable distance have become “infected” with a new form within a relatively short time, it has been possible to trace the source of the seeding in successively affected locations.

## 3. Recent Instances of Disappearing Polymorphs and Elusive Crystal Forms

This section describes several of the most (in)famous recent cases of disappearing polymorphs and other crystal forms. In addition, in relation to the sudden and unexpected disappearance of a well-known crystal form, we consider it particularly relevant to describe cases where elusive crystal forms, believed to be non-existent, were prepared.

### 3.1. Ranitidine Hydrochloride

In the early 1970s, James Black at (then) Smith, Kline & French identified the histamine type 2 (H_2_) receptor and from the preparation of a series of H_2_-receptor antagonists developed the first antiulcer drug, cimetidine (Tagamet®), for which he won the 1988 Nobel Prize in Medicine. H_2_-receptor antagonists are among the miracle drugs of the 20th century. Prior to their introduction (and the subsequent entry of proton pumps) there were millions of sufferers of peptic ulcers worldwide with a significant number of fatalities; since their introduction, the surgical procedure for removing peptic ulcers has essentially been eliminated from the modern medical school curriculum.

**Figure 1:**
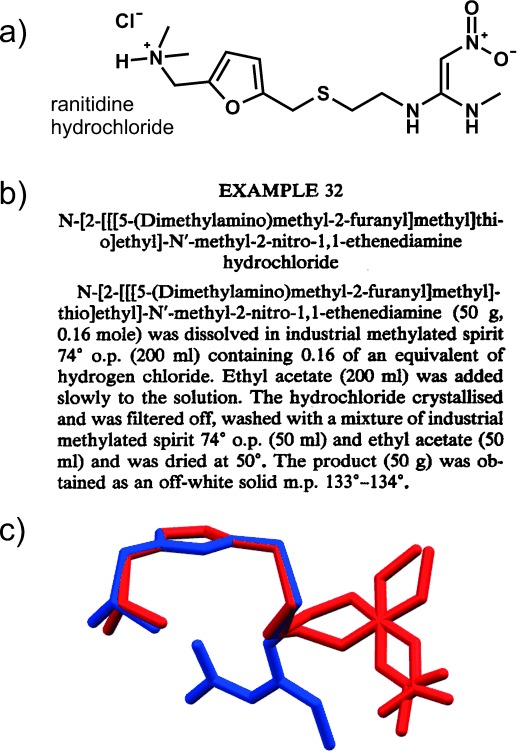
a) Molecular structure of ranitidine hydrochloride. b) Example 32 from patent US 4128658A (“Aminoalkyl furan derivatives”), the apparently straightforward procedure for the preparation of Form 1 of ranitidine hydrochloride. c) Overlay of the ranitidine cation from Form 1 (blue) and Form 2 (red). Form 2 features a disordered nitroethenediamine moiety.

The dramatic success of cimetidine led to industry-wide efforts to develop additional H_2_-receptor antagonists. In 1977, Allen & Hanbury (then a part of Glaxo Group Research, now GSK) developed ranitidine and its hydrochloride (Figure 1 a), for which a US patent was issued in 1978.^25^ The preparation of the hydrochloride following the multistep synthesis of ranitidine base is given in “Example 32” of the patent (Figure 1 b).

Subsequent development of the drug over nearly four years involved batch scale-ups to a multi-kilogram scale in the company’s pilot plant by employing essentially the chemistry described in Example 32.^10^ The batch prepared on April 15, 1980 failed the quality control IR analysis, which exhibited a hitherto unobserved sharp peak at 1045 cm^−1^, and suggested the formation of a new crystal form designated **Form 2**. The subsequent four batches exhibited increasing amounts of **Form 2** and the same process no longer produced the (now designated) **Form 1**. Considerable efforts to revert to the production of **Form 1** by essentially the same process were unsuccessful. Thus, this is clearly a case of a disappearing polymorph. Serendipitously, **Form 2** had considerably improved filtering and drying characteristics which, in addition to the novelty of the new polymorph, formed the basis for a patent application, granted in 1985.^10^ The crystal structures of both forms have been subsequently determined; both forms crystallize in the monoclinic *P*2_1_/*n*, space group, wherein the nitroethenediamine moiety of the ranitidine cations displays different conformations and degrees of disorder (Figure 1 c).^26^–^28^ This is thus also an example of *conformational polymorphism*.^29^

Glaxo launched ranitidine hydrochloride in 1984 as Zantac™ and by 1992 it was the world’s best-selling drug at US$ 3.44 billion per year, when sales for the next largest drug (Bayer’s Adalat Procardia™) were about half that amount.^30^ In accord with the terms of the 1984 Hatch–Waxman Act in the US, by 1990 a number of generic drug companies were planning to enter the market with **Form 1** in anticipation of the 1995 expiration of the **Form 1** patent. Attempts to make **Form 1** were based on carrying out Example 32. As transpired in the course of the subsequent litigations, essentially all of those attempts started with commercial **Form 2**; hence, at the very least **Form 2**—thus seeds of **Form 2**—were present in the environment in which attempts to follow Example 32 were being carried out.

Following numerous attempts to prepare **Form 1** according to Example 32, which led almost exclusively to **Form 2**, the Canadian generic firm Novopharm claimed that Glaxo had never made **Form 1** and sought approval from the Food and Drug Administration (FDA) to market **Form 2**. Glaxo aimed to prevent Novopharm (and others) from entering the market with **Form 2** by suing them for the infringement (actually, virtual infringement under the Hatch–Waxman Act) of their **Form 2** patent. Novopharm admitted infringement of **Form 2**, but argued that the **Form 2** patent was inherently anticipated in the **Form 1** patent, since their attempts to prepare **Form 1** according to Example 32 led to **Form 2**. Novopharm contended that the experimental procedures underpinning the **Form 1** patent were flawed, to which Glaxo responded that the opposition’s experiments were contaminated with seed crystals and hence not a faithful reproduction of Example 32 [Clearly, there were no seeds of **Form 2** anywhere prior to April 15, 1980].

The legal concept of *inheren*cy in the United States implies a consistent result of a process, that is, it must be invariable or inevitable that one obtains the later claimed result to establish inherency. Thus, to support its case against inherent anticipation, in principle Glaxo had to demonstrate that Example 32 did not inevitably or invariably yield **Form 2** but could in fact yield **Form 1**.

To do so, in the course of the August 1993 trial, the notebooks of David Collin, who been the first to prepare ranitidine hydrochloride were examined, cross-examined, and compared to the wording in Example 32. Collin’s notebooks contain three slightly different examples. As is common for a laboratory notebook, the texts are not word-for-word identical nor is any one identical to the language in Example 32, and there was much discussion over the differences and what they would mean to a practicing chemist (one “skilled in the art” in patent lexicon).

In addition, one of Glaxo’s witnesses, Sir Jack Baldwin of the University of Oxford, in 1993 had two of his senior postdoctoral fellows complete the entire synthesis of ranitidine base according to the **Form 1** patent followed by the reproduction of Example 32 using that prepared base. They also obtained **Form 1** three times.

Those six instances of the preparation of **Form 1** according to Example 32 were sufficient to overcome the inherency argument. The **Form 2** patent was found to be valid and Novopharm (and others) were restricted from marketing **Form 2** prior to its anticipated expiration in 2002.

***Legal footnotes.*** A number of litigation cases ensued. It was surprising that Glaxo could no longer make **Form 1** in the original pilot plant, but even so, at the time, the concept of disappearing polymorphs and the role of unintentional seeding were treated with skepticism by those who had no personal experience of the phenomenon. For instance, counsel for Novopharm included the following in his opening statement to the court:^31^

“*There’s also testimony in this case which is under a protective order from a third party pharmaceutical company that did the same thing. They reproduced example 32 and got **Form 2**. So we have six different locations or incidents where example 32 had been reproduced to yield **Form 2**, not **Form 1**. What’s Glaxo’s response to this? Seed crystals, they’re in the air. You can’t see them. You can’t smell them. You can’t taste them. You also can’t detect them but they’re there, and these seed crystals fall out of the sky, and they’re very intelligent because they know when you’re running one of these example 32 experiments. They fall out of the sky and they fall in your reaction beaker and it causes not **Form 1** to be produced, but **Form 2**, and that’s why when we run this experiment today we get the **Form 2** product and not the **Form 1**.*”

“*Well, I submit that if one believes in Santa Claus we might believe in these seed crystals, but if we’re beyond that, we’re not going to believe in these seed crystals, and even if you do, the techniques that were used in these reproductions would, without a doubt, exclude these seed crystals because these seed crystals to survive in the method that has been used for these reproductions have to defy standard chemical principles*”.

Some excerpts from the cross-examination of one of Novopharm’s witnesses regarding seeding:

**Question** (cross-examining attorney): “*I think the issue of seeding is one that I would have expected to come from a crystallographer. Have you made a study of the subject of seeding?*”

**Answer**: “*I’ve found in my experiments that I can’t see any seeding effects*.”

**Question**: “*You found that you can’t see. My question was, have you done a study of the science of seeding which takes in account the myriad of works of those who can see. Have you made such a study?*”

**Answer**: “*I’ve done a literature search to see if a theoretical phenomena (sic) like the hypothetical theory of universal seeding could be found in all of the chemical abstract literature, and the only references I found to something called universal seeding had to do with entries like, universal prevention of fungus on weed seeds by using certain different fungicides. That’s the only type of reference I could come up with when I scanned the chemical literature.*”

**Question**: “……*Now, did you go to any meeting of professional crystallographers or did you consult crystallographers to see whether there was a body of knowledge that you hadn’t found?*”

……**Answer**: “*I reported my negative findings to the [attorneys]*.”

### 3.2. Ritonavir

**Figure 2:**
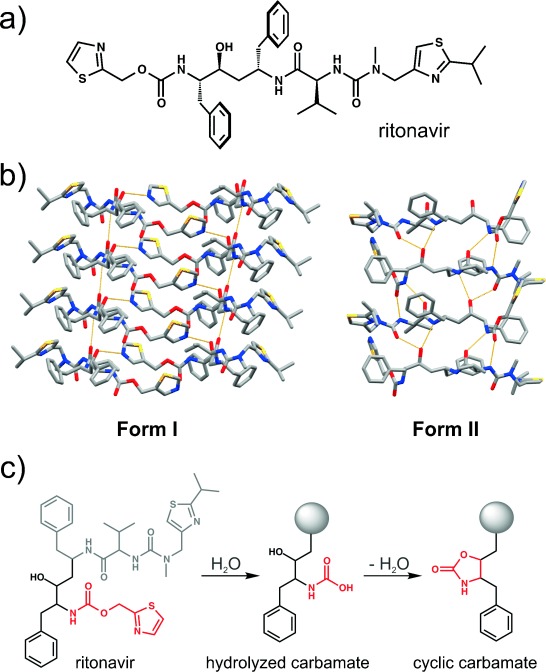
a) Molecular structure of ritonavir. b) Crystal structures of its Form I (left) and Form II (right). c) Formation of the presumed heteronuclear seed of ritonavir Form II through a base-catalyzed reaction.

Perhaps the most notorious recent example of a disappearing polymorph is that of ritonavir (Figure 2 a), an antiviral compound marketed by Abbott Laboratories in 1996 as Norvir™ in the form of semisolid gel capsules for the treatment of the acquired immune deficiency syndrome (AIDS). The capsules were based on the only known crystal form, **Form I**, discovered during the development process. In 1998, however, a new and significantly less-soluble polymorph of ritonavir unexpectedly precipitated out in the semisolid gel capsules, thereby leading to failed dissolution tests for the capsule.^32^, ^33^ Subsequent studies showed that the new form, referred to as **Form II**, exhibited a significantly lower solubility in hydroalcoholic solutions than the marketed **Form I**.^33^ In addition, it was found that **Form II** rendered **Form I** unobtainable in any laboratory to which **Form II** had been introduced. There was even speculation that the conversion of **Form I** into **Form II** in the laboratory was facilitated simply by the presence of an individual who had previous exposure to **Form II** (or the contaminants that were subsequently shown to enable the formation of **Form II**). As a result of these events, ritonavir had to be temporarily withdrawn from the market.^34^

Crystallographic analyses showed that the crystal structure of **Form I** is characterized by ritonavir stacks resembling a β-sheet structure (Figure 2 b).^32^ The structure is sustained by N-H(amide)⋅⋅⋅O(amide) and O-H(hydroxy)⋅⋅⋅N(thiazole) hydrogen bonds (with first-order graph set **N_1_**=*C*(4)*C*11)^35^). The crystal structure of **Form II**, on the other hand, is comprised of heavily hydrogen-bonded one-dimensional ritonavir stacks (Figure 2 b).^36^ Each molecule in the stack is hydrogen bonded to two other molecules through a total of eight N-H(amide)⋅⋅⋅O(amide), N-H(amide)⋅⋅⋅O(hydroxy), and O-H(hydroxy)⋅⋅⋅O(amide) hydrogen bonds (**N_1_**=*C*(6)*C*(9)*C*(11)*C*(12)).^32^, ^35^ The higher calculated crystal density of **Form I** suggested it is also the more stable crystal form.^37^ In addition, a survey of the Cambridge Structural Database (CSD) indicated that **Form I** ritonavir exhibits a statistically more favorable conformation of the carbamate moiety.^32^ The analysis was in agreement with an NMR study in solution that revealed the existence of two conformers in solution in a ratio of roughly 99:1. The conformers could not be unambiguously resolved as **Forms I** and **II**, but it was noted that the observed 99:1 relationship of the two conformers is consistent with the initial discovery of a single polymorph, that is, **Form I**.^32^ The higher stability of **Form II** was, in the end, attributed to the formation of a hydrogen-bond pattern wherein, unlike in **Form I**, “*all of the strong hydrogen bond donors and acceptors have been satisfied”*.^33^ This argument is consistent with the observation that **Form II** has a higher melting point and heat of fusion (ca. 125 °C, 87.8 J g^−1^) than **Form I** (ca. 122 °C, 78.2 J g^−1^).^33^

A recent logistic regression hydrogen-bonding propensity study involving **Forms I** and **II** (using the CSD as data source) reported that the kinetically more favored **Form I** displays a statistically doubtful hydrogen-bond pattern. Specifically, it was found that **Form I** entails statistically improbable hydroxy-thiazoyl and ureido-ureido interactions—despite the hydrogen-bond donor’s and acceptor’s availability for the realization of high-propensity hydrogen bonds.^38^

The origin of **Form II** was initially unclear, as it was established that ritonavir solutions would crystallize as **Form II** only if seeded with **Form II**—even at amounts as low as 1 ppm. Heterogeneous nucleation was identified as a possible cause of the formation of **Form II**. Specifically, it was found that ritonavir degrades in a base-catalyzed reaction to form a carbamate-bearing product (Figure 2 c) structurally related to the conformation of ritonavir in **Form II**.^32^ It was also found that the degradation product forms very rapidly and that, consistent with its greater stability, it exhibits a lower solubility than ritonavir. It was concluded that the degradation product had possibly crystallized out of a ritonavir bulk solution, which had experienced solvent loss, and then acted as a seed for **Form II**.

Eventually, consistent with the closing statement in the 1995 review, extensive studies demonstrated that the crystallization of **Form I** could be achieved under controlled conditions in laboratories that had previously been exposed to **Form II**.^33^ Ritonavir was finally reformulated and approved in 1999 before being placed back on the market.^39^ It was estimated that the company had experienced losses in revenue of over US$ 250 million.^34^

A more recent study attempted a high-throughput polymorph screen of ritonavir to comprehensively explore the compound’s structural diversity. The screen included about 2000 crystallization experiments and resulted in the finding of three new crystal forms in addition to **Forms I** and **II**—namely, a metastable polymorph, a trihydrate, and a formamide solvate.^40^ These findings highlight the necessity of utilizing a variety of crystallization methods, in this particular case, high-throughput screening, combined with carefully designed crystallization experiments for the retrieval of the relevant structures associated with the structural landscape^41^ (also referred to as packing landscape^13^) of a molecule.

***Public relations footnote**.* As noted, Abbott’s initial encounter with **Form II** and its inability to produce **Form I** led to the disappearance of the drug Norvir*®* from the market, leaving tens of thousands of AIDS patients without medication. This led to a serious public relations problem for Abbott. To allay public concern, the company held a number of interviews and press conferences, at which senior Abbot officials appeared in order to answer questions. The transcripts were originally published on the website^42^ of the International Association of Physicians in AIDS Care, but no longer appear there. Some excerpts vividly portray the situation that can arise when a disappearing polymorph is encountered:

“*There was no gradual trend. Something occurred that caused the new form to occur……There was no early warning*.”

“*We, quite honestly, have not been able to pinpoint the precise conditions which led to the appearance of the new crystal form. We now know that the new form is, in fact, more stable than the earlier form, so nature would appear to favor it……**Form II** is new.*”

“*We did not know how to detect the new form. We did not know how to test for it. We did not know what caused it. We didn’t know how to prevent it. And we kept asking the question, why now?……We did not know the physical properties of the new form……We did not know how to clean it, and we did not know how to get rid of it.*”

“*……our initial activities were directed toward eliminating **Form II** from our environment. Then we finally accepted that we could not get rid of **Form II**. Then our subsequent activities were directed to figuring out how to live in a **Form II** world*.”

“*This is why all of us at Abbott have been working extremely hard throughout the summer [of 1998], often around the clock, and sometimes never going home at night. We have been here seven days a week and we will continue to do so. We have cancelled vacations and asked our families for their understanding and support. This is not an issue that we take lightly.*”

“*There were several sub-teams of three to 600 people per team working full time in different areas. We also called on as many resources as we could.*”

“*We tried everything. We conducted countless experiments. We reconditioned our facilities. We rebuilt facilities and new lines. We looked at alternative sites. We visited a number of [other] organizations around the world……to see if we could start clean in a new environment free of **Form II**.*”

“*In a matter of weeks—maybe five or six weeks, every place the product was became contaminated with **Form II** crystals.*”

**Question:** “*You are a large multinational company. Your scientists are obviously smart. How could this happen?*”

**Answer**: “*A company’s size and the collective IQs of their scientists have no relationship to this problem……This obviously has not happened to every drug. But it has happened to other drugs.*”

### 3.3. Paroxetine Hydrochloride

**Figure 3:**
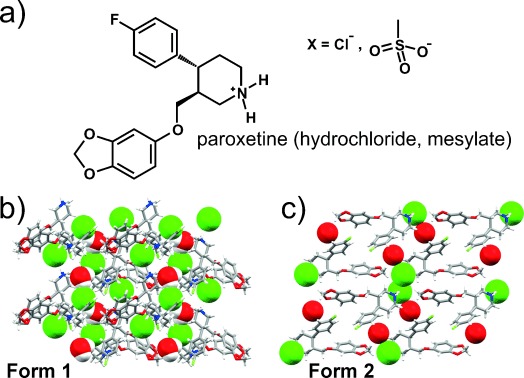
a) Molecular structure of the paroxetine cation. b) Crystal structure of Form 1 viewed along the crystallographic *c* axis. c) Crystal structure of Form 2 viewed along the crystallographic *b* axis. The positions of the water hydrogen atoms in Form 1 were not determined (green spheres: chloride anions; red spheres: water molecules).

Paroxetine hydrochloride is a serotonin re-uptake inhibitor used for the treatment of depression. The chemical compound paroxetine was initially developed by the Danish company Ferrosan in the 1970s. Beecham (now part of GlaxoSmithKline, GSK) purchased the rights to paroxetine in 1980 and undertook development of the hydrochloride salt of paroxetine as a drug product. Beecham developed a process that produced paroxetine hydrochloride in a crystalline form that was later referred to as the “anhydrate” crystalline form. Late in 1984, however, in the course of pilot plant scale-up, the “hemihydrate” crystalline form suddenly appeared at two Beecham sites in the UK within a few weeks of each other. The new hemihydrate form was designated **Form 1** and the previously existing anhydrate was labeled **Form 2** (Figure 3).

The hemihydrate was not hygroscopic and exhibited handling properties superior to those of the anhydrate. In 1986 GSK applied in the US for a patent on the hemihydrate, which was granted in 1988.^43^ Paroxetine hydrochloride was finally marketed as the hemihydrate form in 1993 under the name Paxil®.^10^, ^44^

During the 1980s in the course of the development of the compound for eventual launch, Beecham investigated the properties of both the anhydrate and hemihydrate. They determined that in the presence of water (or humidity) the anhydrate undergoes a conversion into the hemihydrate, a process that is accelerated by temperature, pressure, and the presence of seeds of the hemihydrate.^45^ In Beecham’s experience, it was difficult to avoid the conversion of the anhydrate to hemihydrate in the presence of water or humidity in a facility seeded with hemihydrate.

In 1998, Apotex, a Canadian generic drug manufacturer, filed an Abbreviated New Drug Application (ANDA) with the FDA to market a generic version of the off-patent anhydrous paroxetine hydrochloride (**Form 2**). The hemihydrate patent would expire in 2006. Again, under the terms of the Hatch–Waxman Act GSK opposed the Apotex request, arguing that the anhydrous form (**Form 2**) would convert into the hemihydrate (**Form 1**). GSK’s argument was based, in part, on evidence that Apotex had begun development of its anhydrous product by bringing the hemihydrate into its manufacturing facility, thus providing the seeds that had been shown to be a factor in the conversion. In addition, there was contact with water in the manufacturing of the active pharmaceutical ingredient (API), in the formulation, and in the production of the final water-based coating process of the pill, as well as the use of pressure in the last processing step.

A trial was held in February, 2003 in the US Federal Court, Chicago, with Judge Richard Posner presiding.^46^ Judge Posner is one of the most cited judges in the history of the US federal courts, having written over 2500 published decisions, and as a Professor of Law at the University of Chicago has published nearly 40 books.

GSK’s assertion that Apotex would infringe the hemihydrate patent was based on GSK and Apotex documents showing that many of Apotex’s anhydrate batches had exhibited evidence of conversion: there were batches of anhydrate that converted almost entirely into hemihydrate when stored at 40 °C and 75 % humidity within one month. This Apotex experience was bolstered by the results of GSK’s testing of Apotex’s API and its formulated tablets. Furthermore, Apotex’s specification for release of bulk material was based on a visual method of comparing spectral data that could not detect less than 5–8 % of infringing hemihydrate in the bulk API.

In its defense, Apotex argued that seeding is “junk science”, not widely accepted in the scientific community and that the mechanism of the role of seeds and the conversion is not understood. Moreover, it claimed that the supplier of the bulk API had improved the process to avoid contact with water and was storing the material in improved storage bags, less permeable to water.

In response, GSK argued that Apotex produced the tablets under conditions of normal humidity and sprayed the tablets with an 88 % water-based aqueous coating.

Judge Posner rejected Apotex’s contentions concerning seeding, stating “*that there is no scientific basis for believing that seeding occurs……is obviously wrong*.” In his ruling on the case he found that Apotex’s anhydrate converts into the hemihydrate (in accord with the earlier publication by SmithKline Beecham scientists) and that it “*may continue until it reaches 100 per cent*.” He also found that Apotex’s limit of detection of the hemihydrate in an allegedly anhydrous material was 5–8 %, but he did not rely on this finding in determining whether Apotex was infringing the GSK patent.

Nevertheless, Judge Posner ultimately ruled in Apotex’s favor. Claim 1 of GSK’s hemihydrate patent recited “Crystalline paroxetine hydrochloride hemihydrate”. Judge Posner ruled that this was valid, but that Apotex’s product would not likely infringe the patent because Apotex would not be making it intentionally, and not *“in any commercially significant quantity”*. Judge Posner interpreted Claim 1 as limited to only “*commercially significant amounts of hemihydrate*” and explained that the concentration would have to be in the “*high double digits to contribute any commercial value*”. He further stated that GSK had not established that Apotex would be marketing material with high double-digit concentrations of hemihydrate and that Apotex would not benefit monetarily from the hemihydrate.

Judge Posner thus found the patent valid, but also that Apotex would not infringe it. The case was appealed to the US Federal Circuit Court, which handles all patent appeals in the United States. The Federal Circuit ruled in favor of Apotex, but for different reasons than those invoked by Posner. The Federal Circuit also found that Judge Posner’s claim construction was incorrect, and that the claim properly covered *any* amount of hemihydrate. The Federal Circuit, however, reasoned that Claim 1 must, therefore, be invalid for inherency because if anhydrate converted into the hemihydrate now, it must have converted into hemihydrate in at least small amounts in the prior art. The US Supreme Court refused to hear the case.

Many aspects of those rulings, and the way they were subsequently reported in the trade press, deal with important aspects of disappearing polymorphs and seeding. Details are provided in the Addendum of this Review.

A very recent study demonstrates that it is not possible to claim that all the probable crystal forms of a compound have been found or even fully characterized. Pina et al. showed that **Form 2**, which had initially been described as a hygroscopic anhydrate, was in fact a nonstoichiometric hydrate,^47^ which dehydrates and rehydrates very easily, despite a lack of continuous channels in the crystal lattice. The higher stability of **Form 1** was justified by a higher number of hydrogen bonds being involved in retaining the water molecules in the crystal lattice,^47^ although no quantitative estimates of the relative stabilities were calculated.

### 3.4. Paroxetine Methane Sulfonate (Paroxetine Mesylate)

The market success of paroxetine hydrochloride (worldwide sales of US$ 3.2 billion in 2001) led many pharmaceutical companies to search for additional crystal forms. One approach is to prepare a different salt. In the mid-1990s SmithKline Beecham (now GSK) in the UK and Synthon in the Netherlands independently succeeded in making the mesylate (methanesulfonate) salt of paroxetine. Considering the nature of the chemistry, it is not surprising that the procedure leading to the salt is similar in the two patents. The first US patent was issued to Synthon on 23.02.1999. Another US patent was issued to SmithKline Beecham nearly 15 months later on 16.05.2000.

How could two patents be issued for the same salt? One clear possibility is that they are two different crystal forms of the same salt, which should be discernible from the analytical data presented in the patent. Table 1 contains a comparison of the relative data from the two patents. Clearly, the only data that can be compared are those from the IR spectra. The IR data from the two patents are given in Table 2. Comparison of the two peak lists indicates that they do not characterize the same crystal form. In the course of litigation it became apparent that every reported synthesis of paroxetine mesylate (since the issuance of the Synthon patent) has yielded the peaks found in the SmithKline Beecham patent. There are only two explanations for this situation: 1) the Synthon form is a disappearing polymorph, having been prepared and characterized at least once, and subsequent preparations and crystallizations led to the SmithKline Beecham form, or 2) the Synthon data are in error.

**Table 1:** Summary of analytical data from two patents on paroxetine mesylate.

	Synthon	SmithKline Beecham
melting point	142–144 °C	143–146 °C
		
XRPD	–	peak list
		
IR spectrum (peak list)	KBr pellet: 18 peaks in specifications	8 peaks^[a]^ in Claim 1 (±4 cm^−1^)
		
NMR	solution	–

[a] Nujol: 12, 18, or 35 peaks; ATR: 32 peaks; KBr: 35 peaks.

**Table 2:** Comparison the IR peak lists reported in the two patents on paroxetine mesylates [cm^−1^].

Synthon	SmithKline Beecham
3023, 2900, 2869, 2577, 1615, 1515, 1500, 1469, 1208, 1169, 1100, 1038, 962, 931, 838, 777, 546, 531	1603, 1194, 1045, 946, 830, 601, 554, 539 (±4 cm^−1^)

A series of litigations on the issues associated with the paroxetine mesylate reported in these two patents (and their European equivalents) failed to achieve a legal consensus on the issue. Some of those litigations are summarized in Table 3.

**Table 3:** Summary of the litigations between SmithKline Beecham Corp. and Synthon Pharmaceuticals Ltd.

Forum	Case No.	Disposition	Patents Disputed
United States Middle District of North Carolina (M.D.N.C.)	1:00CV01179	Settlement filed Dec. 30, 2003	**US Pat. No. 4,721,723**, issued Jan. 26, 1988, titled “Anti- Depressant Crystalline Paroxetine Hydrochloride Hemihydrate” **US Pat. No. 6,063,927**, issued May 16, 2000, titled “Paroxetine Derivatives” **US Pat. No. 6,113,944**, issued Sept. 5, 2000, titled “Paroxetine Tablets and Process to Prepare Them”
			
The Hague	T 0885/02–3.3.1.	Ruling for Synthon, European Patent revoked	**EP0970955**, published Dec. 1, 2000, titled “Paroxetine Menthanesulfonate”
			
House of Lords	[2005] UKHL 59	Ruling for Synthon, UK patent invalid	**UK Pat. No. 2336364**, filed on Apr. 23, 1999, published May 10, 2000, titled “Paroxetine Salt”

Although not reported in the literature, the paroxetine mesylate case is an outstanding example that underpins the necessity to thoroughly investigate, characterize, and document APIs and speciality chemicals.

### 3.5. Rotigotine

**Figure 4:**
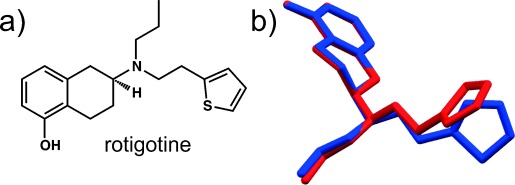
a) Molecular structure of rotigotine and b) overlay of rotigotine molecules extracted from the crystal structures of Forms I and II (shown in red and blue, respectively). Only the major occupation site of the disordered thiophene moiety is shown.

Rotigotine (Figure 4 a) is a non-ergot-derived dopamine agonist initially prescribed for the treatment of Parkinson’s disease, and later approved for moderate-to-severe cases of restless-legs syndrome. It is marketed by UCB under the name Neupro®, and administered through a transdermal patch to minimize the unpleasant side effects of the drug.^48^

Neupro® was approved by the European Medicines Agency (EMEA) for use in Europe and then by the FDA for the US market in 2007. In 2008, a previously unknown and thermodynamically more stable polymorph emerged in the Neupro® patches, in the form of “snow-like crystals”. The new polymorph was unanticipated and unexpected, as the drug had been established since the 1980s and no polymorphism had been observed during drug development or thereafter.^48^, ^49^ While the new polymorph (**Form II**) exhibited no reduction in efficacy, physicians, distributors, pharmacists, and patients were advised to refrigerate their Neupro® stocks, since refrigerated storage significantly reduced crystallization rates. UCB continued to supply Neupro® in Europe, but specific batches were recalled and replaced by batches that were refrigerated immediately after manufacture. While there was “only” a minor disruption in Neupro® supply in Europe, the situation was much more serious in the US, where Neupro® became temporarily unavailable. After the FDA recommended reformulation of the drug, UCB’s new Neupro® formulation that did not require refrigeration was approved by the agency in 2012.

The origin of the more stable **Form II** of rotigotine is not known, but it is reasonable to speculate that the polymorphic transformation was suddenly triggered by an event, or an impurity, in the patch or the drug itself. The initially observed and long-known polymorph (**Form I**) crystallizes in the tetragonal *P*4_3_ space group,^50^ while **Form II** crystallizes in the orthorhombic *P*2_1_2_1_2_1_ space group.^51^ Both polymorphs feature disordered thiophene moieties and similar hydrogen-bonding patterns of one-dimensional zigzag chains of O-H⋅⋅⋅N hydrogen bonds [**N_1_**=*C*(8)^35^]. However, **Form II** is more dense (and thus likely more stable, according to the Burger–Ramberger density rule^37^) and accompanied by a conformational change caused by the adjustment of the torsion angle between the thiophene and alkyl moieties.

### 3.6. DMP 543

**Figure 5:**
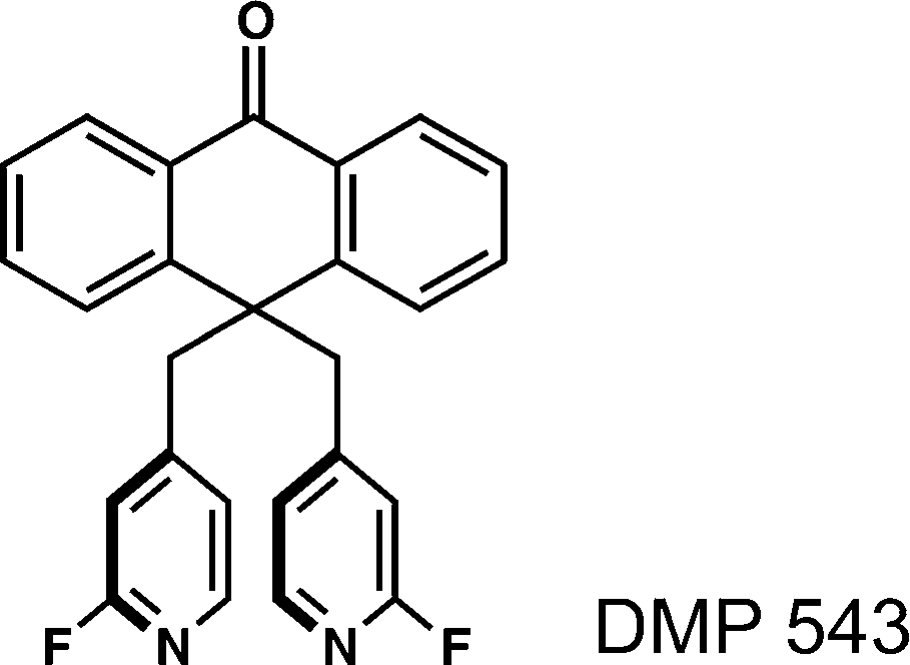
Molecular structure of DMP 543.

DuPont entered the development of pharmaceutical agents for the treatment of Alzheimer’s disease in the 1980s. Extensive studies finally resulted in the development of DMP 543—an acetylcholine release enhancer with the desired potency, plasma duration, and brain penetration properties (Figure 5). It became evident in the early stage of the drug’s development that DMP 543 was very susceptible to polymorphism, as 17 polymorphs were produced and characterized by powder X-ray diffraction.^52^ The authors attributed the ability of DMP 543 to form such a large number of polymorphs to the conformational flexibility of its pyridyl groups, although it has recently been determined that there is no statistical correlation between molecular flexibility and the tendency to polymorphism.^29^ It was found that some polymorphs interconvert easily on heating. But more intriguingly, apparently identical recrystallization conditions would not always lead to the formation of the same polymorph. A robust method for the crystallization of a single polymorph was finally established using an ethyl acetate/heptane solvent mixture. The first process batch, however, yielded a previously unknown polymorph (**#18**). Although this procedure initially appeared robust, researchers were concerned that the preparation of the solvent mixture might not be reproducible and that differential solvent evaporation rates could, in future experiments, trigger the formation of other new and possibly unwanted solid forms. This led to the decision to produce a crystallization procedure based on a single solvent. It was then found that isopropanol reliably produced another new polymorph (**#19**, **Form A**) in high yields, and this polymorph was finally chosen for further development.

After the initial batch of **Form A** was prepared, the synthetic procedure for DMP 543 was refined, and this modified procedure was utilized to prepare the second batch. The synthesis of the second batch began in Deepwater, NJ (USA), but was completed at a different location, namely the Merck Frosst Centre in Dorval, Quebec, where the clinical trials were intended to be conducted. During the course of the synthesis, the anthrone alkylating agent had to be purified three times (by two recrystallizations and one chromatographic purification) before the compound was finally recrystallized from isopropanol to achieve 98.5 % purity, which was the lowest purity grade specification acceptable for clinical trials. A second recrystallization of the solid, however, resulted (to everybody’s surprise) in the formation of yet another new polymorph (**#20**, **Form B**). Three subsequent recrystallizations were performed using the new form, utilizing seeds of **Form A** with the hope of producing a large batch of **Form A**. All three recrystallization experiments yielded **Form B**. Attempts to prepare **Form A** at DuPont in Deepwater resulted exclusively in the formation of **Form B**—very shortly after **Form B** was discovered at the Merck Frosst site in Dorval.^52^, ^53^ DuPont was never again able to produce **Form A. Form B** turned out to be the most stable of all polymorphs according to thermal analysis and was, therefore, selected as the preferred crystalline form of DMP 543 for production.^53^ Although no new polymorphs were found in the following five years, DMP 543 was never commercialized.

### 3.7. LAB687

**Figure 6:**
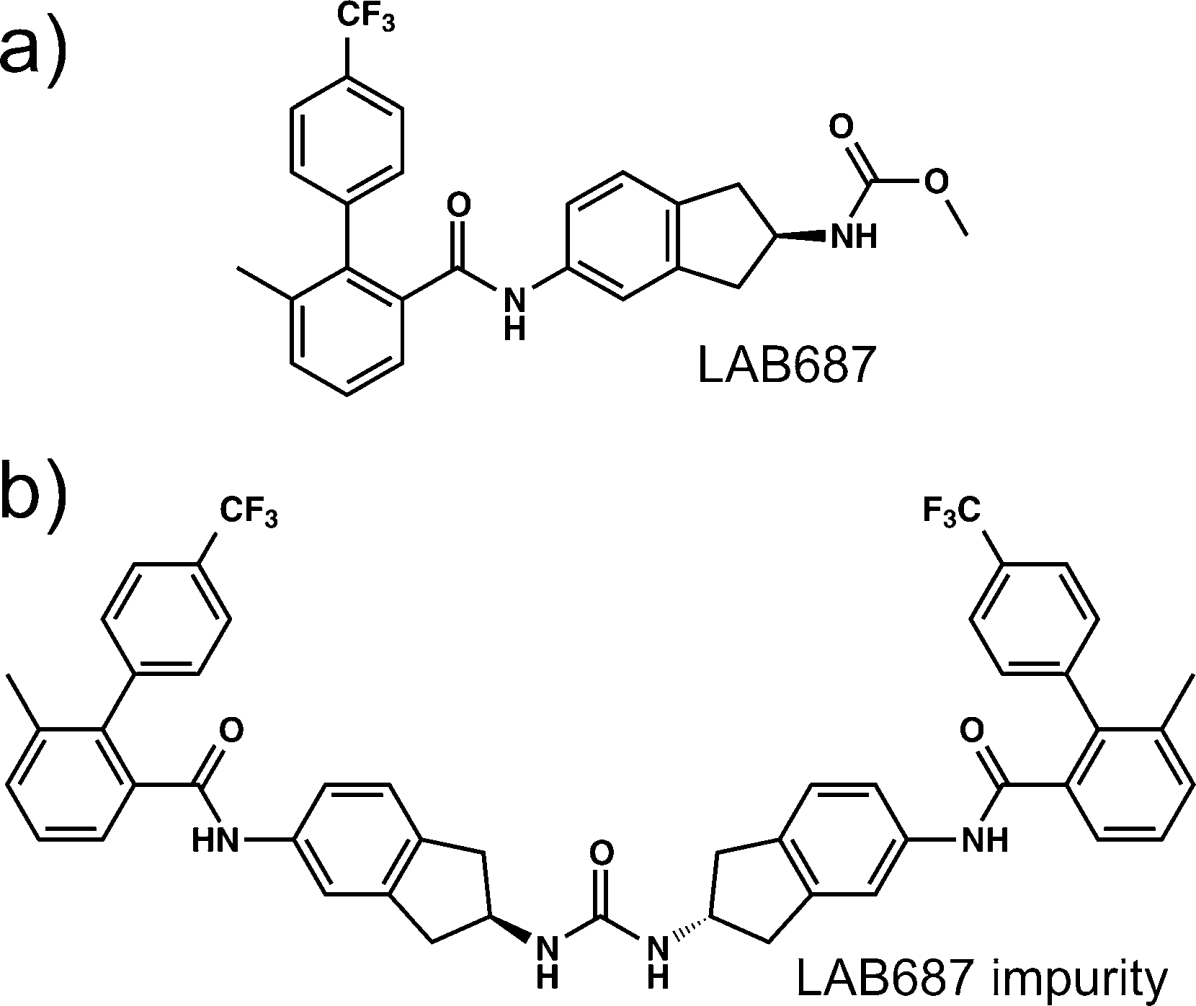
a) Molecular structure of LAB687, and b) the dimeric urea impurity believed to be responsible for the formation of LAB687 Form D.

Another extraordinary account of the unpredictability of polymorphism comes from Novartis, and involves a compound internally identified as LAB687 (Figure 6 a),^54^ an inhibitor of the microsomal triglyceride transfer protein developed for the control of triglyceride and LDL-cholesterol levels.^55^ Two polymorphs were found during drug development: the original synthetic route yielded **Form B**, while a gram-scale synthesis based on a different synthetic procedure led to the formation of **Form A**.

A subsequent polymorph screen was conducted using a 98.9 % pure batch of **Form A**, which yielded a third polymorph (**Form C**), as well as a toluene solvate. Two solvates based on heptane methylcyclohexane were then later discovered during the development of seeding-based crystallization procedures for the reproducible larger-scale (100 g) synthesis of **Forms A** and **C.** Although **Forms A** and **C** were found to have similar intrinsic solubilities and physical stabilities, **Form C** was selected for further development because it had better filtration and flow properties than **Form A**. Remarkably, once **Forms A** and **C** were discovered, **Form B** could not be reproduced. A new polymorph, **Form D**, unexpectedly appeared when the crystallization process for **Form C** was scaled up for a multi-kilogram synthesis. Oddly, once **Form D** emerged, **Forms A** and **C** could no longer be prepared. It was assumed that **Form D** appeared due to a change in the impurity profile of the LAB687 batches during the implementation of a new and more-efficient phase I synthetic route for one of the LAB687 intermediates. Indeed, this route led to the formation of a dimeric urea byproduct (i.e. an impurity, Figure 6 b) that was not present in batches obtained using other synthetic routes, although no direct correlation could be determined between the formation of this impurity and the appearance of **Form D**.

### 3.8. Sulfathiazole

**Figure 7:**
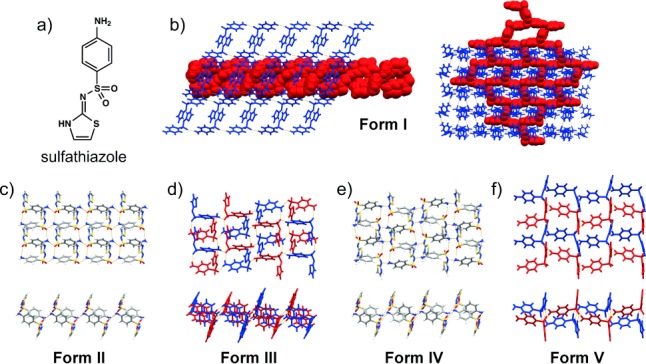
a) Molecular structure of sulfathiazole. b) Crystal structures of Form I showing how sulfathiazole builds a three-dimensional hydrogen-bonded network (left) that is interpenetrated by a two-dimensional hydrogen-bonded network (right). The two networks are built from crystallographically independent molecules (colored red and blue). c–f) Crystal structures of Forms II–V showing the formation of two-dimensional hydrogen-bonded networks. The two-dimensional networks in Forms III and V (shown in (d) and (f), respectively) are sustained by two crystallographically independent sulfathiazole molecules (colored red and blue).

The unexpected formation of polymorphs of the antimicrobial compound sulfathiazole (Figure 7 a) is also triggered by impurities or reaction byproducts. Five polymorphs of sulfathiazole have so far been identified (Figure 7 b–f)^56^–^60^ in addition to over one hundred solvates.^61^ The solid-state chemistry of the sulfathiazole polymorphs has been thoroughly analyzed^62^, ^63^ and numerous research groups reported different methods for the preparation of each polymorph.^62^ It is, however, also reported that some methods cannot be used with confidence for the formation of phase-pure batches of the targeted sulfathiazole polymorphs, since different research groups reported different outcomes using the same crystallization conditions.

A recent review^64^ addressed the widespread belief that a particular sulfathiazole polymorph is *consistently* accessible from a given solvent, and suggested that this view was in fact misleading. The author shared his personal experience from over 2000 crystallization experiments involving sulfathiazole and revealed that all five known polymorphs can be obtained from nearly every solvent used in his comprehensive studies. It was also stated that the polymorphs are enantiotropically related and that the solid’s treatment after crystallization is most critical in determining the polymorphic outcome. Indeed, the sensitivity of the sulfathiazole solid towards post-crystallization treatment is in keeping with the inconsistent results reported in the literature, which shows that the same (or very similar) crystallization conditions can lead to the formation of different sulfathiazole polymorphs.

Despite the inconsistencies in the reported crystallization outcomes, it has been established that the crystallization of sulfathiazole from water follows Ostwald’s rule of stages,^12^ whereby the least-stable polymorph (**Form I**) appears first, and transforms into **Forms II** and **III** before the transformation ends with the appearance of the thermodynamically stable **Form IV**.^63^, ^65^ Notably, it has also been demonstrated that the transformation (and disappearance) of the metastable **Form I** can be suppressed by the presence of impurities in the crystallization solution. More specifically, a compound that forms as a byproduct of the sulfathiazole synthesis (i.e. ethamidosulfathiazole) was used to show that concentrations as low as 10 mol % stabilize metastable **Form I**, while amounts of 0.5–1.0 mol % yielded a mixture of the four **Forms I**–**IV**. Pure solutions, as well as those with an impurity content of 0.01 mol %, were shown to yield **Form** **IV**.^65^ The ability of ethamidosulfathiazole to stabilize **Form I** was attributed to its capacity to integrate into the growing crystal faces of **Form I** without disrupting crystal growth. The growth of **Forms II**, **III**, and **IV**, on the other hand, becomes inhibited once ethamidosulfathiazole becomes attached to their growing crystal faces.

Although sulfathiazole polymorphs do not tend to “disappear for good” as claimed for some other polymorphs described in this Review, the sulfathiazole system seems unique because of its complexity and sensitivity, and highlights the need to understand how byproducts obtained from the synthesis of a target compound can profoundly affect its solid-state chemistry.

### 3.9. Progesterone

**Figure 8:**
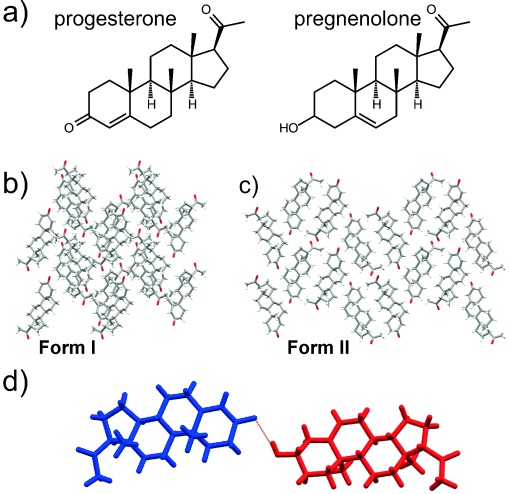
a) Molecular structures of progesterone and pregnenolone. b) Crystal structure of Form I of progesterone. c) Crystal structure of Form II of progesterone. d) Crystal structure of the 1:1 progesterone:pregnenolone cocrystal (progesterone blue, pregnenolone red). Crystal structures in (b) and (c) are viewed along the crystallographic *a* axis.

Crystal structure prediction (CSP) has enjoyed a decade of increasing success rates and an expanding range of applications in the study of molecules of increasing complexity.^66^, ^67^ A recent study suggested that progesterone (Figure 8 a) may be a useful model compound with which to test advances in CSP, as a suitable “real” system of pharmaceutical interest.^68^ Steroids are the basis of fundamental hormones and many drugs; they have rigid structures, and progesterone is a relatively simple example of a steroid. Beyond that, steroids are generally relatively easy to crystallize and they often exhibit polymorphism. Progesterone was chosen as a model compound for CSP based on the above criteria, with a supposedly well-understood polymorphic system documented in the scientific literature stretching back 70 years.

The predictive studies correctly identified the two known polymorphs (**Forms I**^69^ and **II**,^70^ Figure 8 b,c) in enantiomorphic space groups. CSP studies also strongly indicated that there were a number of more-stable centrosymmetric structures with one at the global minimum. This lowest-energy structure was ultimately crystallized by mixing natural (*nat*) progesterone with its enantiomer *ent*-progesterone. When this work was initiated, centrosymmetric structures were not considered because they do not exist naturally for this chiral molecule, but the results from the CSP studies were so compelling that the experimental search for a racemic structure was undertaken. During the course of this study it became apparent that the metastable low-melting-point **Form II** of *nat*-progesterone was an example of a disappearing polymorph, as attempts to make it soon failed and attempted crystallizations became erratic in their polymorphic outcome. Attempts were then made to template progesterone with a structurally related steroid, pregnenolone (Figure 8 a), which resulted in the successful preparation of **Form II** along with the concomitant formation of a 1:1 progesterone: pregnenolone cocrystal.^71^ The metastable form produced by these means was stable for periods ranging from hours to two or three months!

About this time it transpired that the University of Innsbruck had samples of both polymorphs in an archive. Their metastable polymorph had not transformed in over 50 years! The chemical analysis of the long-lasting **Form II** sample revealed impurities that were not present in the sample of **Form I**, thus highlighting the potential role of contaminants/additives in stabilizing metastable crystal forms. Attempts were made to analyze the impurity profiles associated with the archived samples, with a view to doping crystallizations involving modern much purer commercial material. These were unsuccessful owing to the complexity of the impurity profiles associated with the old samples.^72^

### 3.10. The Elusive Form II of Aspirin

**Figure 9:**
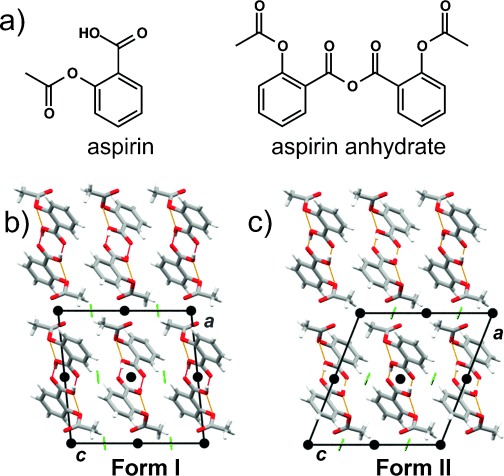
a) Molecular structures of aspirin and the aspirin anhydrate. Crystal structures of b) Form I and c) Form II. The crystal structures in (b) and (c) are viewed along the crystallographic *b* axis. The inversion centers are represented with black spheres, while the 2_1_ screw axes are depicted using green arrows.

Aspirin (acetylsalicylic acid, Figure 9 a) is a widely used analgesic, and its antiplatelet activity makes it a commonly prescribed long-term preventative agent for reducing the risk of heart attacks and strokes.^73^ Although first synthesized more than 150 years ago, only one polymorph^74^ of aspirin (**Form I**) was known until 2005. The potential polymorphism of aspirin was extensively studied and debated in the 1960s and 1970s,^75^ but the existence of another polymorph was not definitely established with certainty.^76^ A study from the early 1980s reported that aspirin, if crystallized in the presence of aspirin anhydride (Figure 9 a), exhibits the characteristic aspirin **Form I** diffractogram, along with weak additional peaks.^77^ It was concluded that these are not likely to correspond to a new aspirin polymorph, but rather belonged to an impurity in the form of a cocrystal or solid solution composed of aspirin and salicylic acid.^77^, ^78^ It took more than 30 years to discover that these “impurity” peaks are actually related to another polymorph, **Form II**.

A computational study published in 2004 predicted that the known crystal **Form I** is the most stable, but it also predicted the existence of a second polymorph that exhibits virtually the same crystal lattice energy as the known form.^79^ The elusiveness of this polymorph was ascribed to its predicted low shear elastic constant, which suggests that the polymorph exhibits an energetic low barrier to transformation into **Form I**. A later study concerning aspirin cocrystals led to the discovery of the new polymorph, **Form II**.^80^ The **Form II** solid was obtained in the course of an attempt to cocrystallize aspirin with levetiracetam in a 1:1 ratio, and although its structure was derived from crystallographic data of lower quality,^80^, ^81^ it was in good agreement with the computationally predicted crystal structure of the elusive low-energy polymorph. **Forms I** and **II** of aspirin are structurally (and energetically) very similar.^79^ Both forms feature centrosymmetric aspirin dimers held together by the carboxylic acid homosynthon. The dimers form two-dimensional layers parallel to the crystallographic *c* axis. The two forms differ in the relative positions of the neighboring dimer layers, as well as the symmetry elements between them (Figure 9 b,c).

Soon after the initial discovery of **Form II**, two studies provided evidence that the initially reported **Form II** is in fact an inter-grown phase^82^ containing domains with structural features corresponding to both **Forms I** and **II**.^83^, ^84^ It should be noted that no solid exhibiting the structural features of **Form II**­ *alone* could be isolated at that point. It was also reported that the inter-grown phase could only *occasionally* be prepared by recrystallization of freshly synthesized aspirin from acetonitrile or tetrahydrofuran. Interestingly, commercial samples crystallized under the same conditions only yielded crystals of **Form I**. It was then discovered that the diffractograms of the inter-grown crystals displayed weak peaks belonging to the aspirin anhydrate, thus indicating that the anhydrate might have played a significant role in the formation of the inter-grown phase.^78^ Further investigations demonstrated that the inter-grown crystals can be *regularly* prepared using aspirin anhydrate as seed material. It was also shown that seed quantities of up to 10 wt % yield inter-grown crystals with a substantial presence of **Form II** domains. Finally, the seeding experiments have also established that *phase-pure* batches of the elusive **Form II** can be reliably prepared if aspirin solutions are seeded with 15 wt % of the anhydrate.^78^

### 3.11. The Elusive (caffeine)⋅(benzoic acid) Cocrystal

Recently, molecular cocrystals have been attracting the attention of pharmaceutical and materials scientists, primarily because of their potential ability to alter the physicochemical properties of molecular compounds^85^–^97^ while maintaining the pharmaceutical activity of the active pharmaceutical ingredient. This deepened interest in cocrystals has led to the development of increasingly sophisticated crystallization techniques,^98^, ^99^ which are generally used during cocrystal screening in an integrated fashion in the search for new (co)crystal forms of a drug candidate. When cocrystallization attempts fail, it is difficult to know whether the failure was due to poorly chosen experimental conditions or because the cocrystal simply cannot form based on thermodynamic considerations, that is, the cocrystal lattice energy is higher than the lattice energy of the cocrystal components.

Caffeine is one of the most utilized model compounds in studies of pharmaceutical cocrystals, and has been shown to engage in cocrystallization with a wide variety of carboxylic acids;^100^, ^101^ despite this, the literature is replete with reports that caffeine does not form a cocrystal with benzoic acid.^102^, ^103^ For example, a recent study describes the efforts of four research groups to prepare the elusive cocrystal using neat grinding, liquid-assisted grinding, and solution-mediated phase transformation.^104^ After all attempts failed, CSP methods^105^, ^106^ were employed to evaluate the potential existence of a 1:1 (caffeine)⋅(benzoic acid) cocrystal, and showed that the formation of the target cocrystal is indeed thermodynamically favored.

The CSP work also aimed to identify appropriate heteronuclear seeds for the cocrystallization of caffeine and benzoic acid. It was presumed that a high kinetic barrier hindered the formation of the cocrystal, and it was proposed that this barrier could be overcome by introducing a heteronuclear seed, which matched the target cocrystal structurally or epitaxially. Fluorinated benzoic acids were used to form cocrystals based on molecular assemblies similar in shape and size to those present in the putative (caffeine)⋅(benzoic acid) cocrystal.

**Figure 10:**
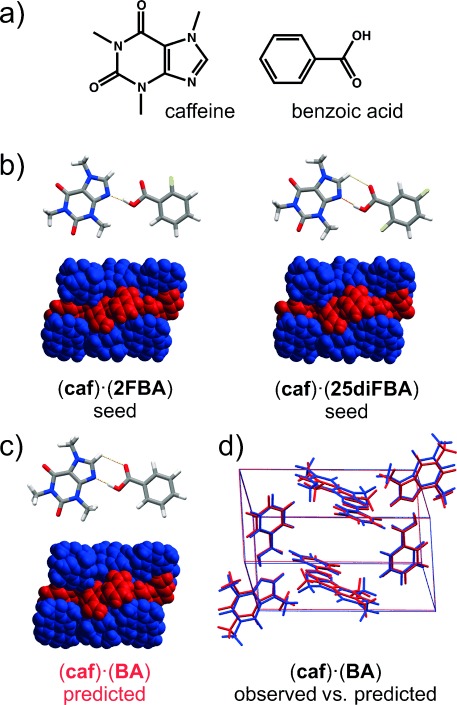
a) Molecular structure of caffeine (caf) and benzoic acid (BA). b) Cocrystals as heteronuclear seeds: cocrystals composed of caffeine (caf), 2-fluorobenzoic acid (2 FBA), and 2,5-difluorobenzoic acid (25 diFBA). c) Predicted lowest-energy crystal structure of (caf)⋅(BA). d) Overlay of the isomorphous lowest-energy predicted and obtained (caf)⋅(BA) cocrystal (red: predicted, blue: observed).

The rationale behind the use of fluorinated benzoic acids lay in the relatively small size difference between the van der Waals radii of hydrogen and fluorine.^107^ The strategy was successful: heteronuclear-seeding experiments yielded the target cocrystal in all four laboratories where “seedless” cocrystallization attempts previously failed (Figure 10). Interestingly, once the heteronuclear seeds were introduced to a laboratory, they—or the seeds of the product cocrystal—continued to act as long-lasting laboratory “contaminants” that enabled cocrystallization even when present at undetectably low levels, an observation consistent with Wiberg’s earlier observation.^22^

The quest for the (caffeine)⋅(benzoic acid) cocrystal demonstrates the utility of CSP calculations in assessing the likelihood of cocrystal formation. At the same time, the study stresses that current cocrystal screening methods need to be improved to eliminate the occurrence of false negative results that could impede the development of functional multicomponent crystalline materials. This study highlights the gaps in our current understanding of the nucleation process of cocrystals and of how laboratory contaminants may affect the outcomes of crystallization experiments.

A related study recently demonstrated that seeding is indeed a practical method of crystallizing anticipated solids that are inaccessible at ambient conditions. Specifically, a monohydrate of the neurotransmitter γ-aminobutyric acid was obtained at high pressure and subsequently recovered at ambient conditions at which its crystallization was unsuccessfully attempted in numerous trials. The high-pressure polymorph could then be consistently used to produce the elusive hydrate at ambient conditions.^108^

## 4. Recovering Disappeared Polymorphs

As noted above, at any particular temperature and pressure, the Gibbs Phase Rule permits the existence of only one thermodynamically stable polymorph of a substance. However, kinetic stability allows the coexistence of more than one form. It is, therefore, possible in principle to prepare and maintain a number of crystal forms at ambient conditions without limitation.

In many of the cases of disappearing polymorphs described above, the form that disappeared was the only one known until a new form appeared—often as a result of the same procedure that previously led to the now absent form. In most cases this means that among the known forms, the new form is the thermodynamically preferred, but not necessarily *the most stable form* under those conditions. Since the disappeared form had been prepared and characterized (often many times and over long periods of time), it must occupy its own definite region in phase space, even if it becomes very difficult to prepare it again. The almost inescapable conclusion from this situation is that the most practical strategy for recovering a disappearing polymorph is to employ kinetic crystallization methods rather than thermodynamic crystallization methods. The dominance of a new form is often also a result of aggressive seeding by that form, thus indicating that those seeds must be assiduously avoided to prepare the disappeared form. The following examples will demonstrate these principles.

One of the first detailed studies of conformational polymorphism involved the study of dimorphic *p*,*p*′-dichlorobenzylideneaniline.^109^, ^110^ Both forms were grown from ethanol solutions. The metastable triclinic form initially crystallized as transparent needles with a 4 Å long axis parallel to the needle axis. Cleaving the crystals perpendicular to the needle axis would induce a transformation to the orthorhombic form that could be detected by an increasing cloudiness of the crystal and a concomitant loss of single crystallinity. Over a relatively short period of time (i.e. a few weeks), as the amount of the orthorhombic form increased in the laboratory, it became increasingly difficult to obtain the triclinic form. The method that finally produced the triclinic form quite consistently (but not always!) was to prepare a maximally saturated solution in boiling ethanol (a beaker seemed to work better than an Erlenmeyer flask) and to immediately place the solution in a closed desiccator freshly charged with CaCl_2_ and minimizing the contact of the solution with the laboratory atmosphere that no doubt contained seeds of the orthorhombic form. This provided a kinetically biased crystallization, combining the high degree of supersaturation with the fairly rapid cooling and the desiccating power of the CaCl_2_. While the triclinic form could be made quite consistently by this method, the eventual solid-to-solid transformation could not be prevented.

We noted above the notion that a laboratory can become seeded with a stable form and render it extremely difficult, if not impossible, to prepare the metastable form in that same laboratory environment. Essentially two solutions are possible—but, again, not always successful—to this situation: 1) move to another laboratory (another distant geographical location may be required) or 2) thoroughly clean the laboratory. We describe an example of each of these solutions.

In 1972, one of us (J.B.) prepared the dimethyl analogue of the dichlorobenzylideneaniline described above, and found the cell constants to be identical to those reported by Bürgi et al. about four years previously.^111^ When we were ready to carry out the crystal structure analysis a few months later the crystals had deteriorated, so the compound was recrystallized using the same ethanol solvent as the previous batch. This resulted in a new polymorph. Over the next two years, numerous syntheses and recrystallizations that followed failed to yield the original crystal form, although a third polymorph did appear.

At that time we were moving into a new laboratory a few kilometers distant from the old one where the original experiments had been done. We hired a new student (by telephone) and instructed her to use newly purchased reagents and virgin glassware in the new lab. The original polymorph was prepared on her first attempt.

The other option to attempt to recover a disappeared polymorph is to cleanse the laboratory of the culpable crystal form. Such a strategy was employed by Nielsen and Borka with benzocaine:picric acid.^112^ The material was used as a pharmacopeial standard in the 1960s. There is a higher melting (162–163 °C) form that was obtained from the lower melting (132 °C) form by drying the latter at 105 °C for at least one hour or by vacuum drying/sublimation. Once the higher melting form was obtained, the lower melting form could no longer be prepared. In the authors’ words: “*As a matter of curiosity, it ought to mentioned that once the stable modification was obtained, the metastable modification could no longer be isolated……It was found that after discarding all samples, washing the equipment and laboratory benches and waiting for 8–12 days, the low-melting modification could be isolated again. This has now been repeated several times in our laboratories*.”^112^

In 1999, we initiated a thorough reinvestigation of this system based very much on hot-stage microscopy,^16^ and in addition to the two 1:1 complexes we confirmed an earlier reported 2:1 complex and a hydrate of a 1:1 complex.^113^, ^114^ In a set of carefully designed experiments we first prepared the low-melting 1:1 form from a saturated aqueous solution at 80 °C, since the hot-stage experiments indicated that the low-melting form is the stable one above this temperature. Thus, we avoided the presence of seeds of the high-melting form, which was subsequently prepared by a non-aqueous gel-diffusion crystallization with both components dissolved in a 3:1 CHCl_3_:CH_3_OH solvent mixture. The 2:1 complex was obtained over a period of four weeks from a 1:1 mixture in isopropyl alcohol. The 1:1 hydrate was obtained after 48 h from a saturated aqueous solution in a sealed virgin flask at 20 °C.

The 1995 review described the joint experience (in Zürich and Beer Sheva) with *p*′-methylchalcone. In the 1920s, the compound was investigated by Weygand for nearly ten years using thermomicroscopy and summarized in a 1929 review.^115^ For many years it competed for the title of “world record holder” for the number of reported polymorphs (albeit lacking structure determinations) of a molecular compound with 13 forms. Weygand distinguished seven modifications (called “main forms”) as monotropically related with a high probability. In our hands, in accord with the finding of Weygand, once the seeds of the most-stable highest melting form (m.p. 75 °C) are present in the laboratory it is virtually impossible to obtain any of the other forms by standard solution crystallization techniques.

It is well known that the polymorphic form may be influenced by the reaction mixture, since the material is crystallizing from a different solution environment.^17^, ^116^ *p*′-Methylchalcone^17^ is prepared by a simple condensation reaction, so that some synthetic conditions—at least the solvent and the temperature—may be readily varied. We carried out the base-catalyzed condensation reaction using the appropriate ketone and aldehyde under nine conditions (three solvents: methanol, ethanol, and 2-propanol; three temperatures: 20 °C, 4 °C, and −13 °C) and obtained five thermodynamically unstable forms directly from the reaction mixture.^17^ As a consequence of their instability, they were not easy to handle or characterize, but we did obtain sufficient evidence to positively identify and distinguish them.

In many of these cases of disappeared polymorphs, if the old form can be obtained, it often transforms to the new, and presumably thermodynamically more stable, form. That situation is by no means universal. For instance, in the case of ranitidine hydrochloride, in spite of the difficulty of preparing **Form 1** in the presence of **Form 2** seeds, the two forms can exist side by side essentially indefinitely, since there is no simple mechanism for the transformation between them.

Recent studies have demonstrated the utility of engineered surfaces^117^, ^118^ and heteronuclear seeds^119^, ^120^ in crystallizing specific polymorphs and discovering new ones. It is possible that such approaches could be utilized to recover polymorphs that had apparently “disappeared”.

The variety of circumstances and conditions for these examples demonstrates that each molecule and each multicrystal form is unique. Recognizing the phenomenon of disappearing polymorphs and learning to overcome and control it requires a combination of considerable skill on the part of the chemist with the acquisition of an intimate familiarity with and understanding of the crystal chemistry of the compound in question.

## 5. Outlook

It should be apparent from the content of this Review that the mere existence of polymorphs and polymorphic transformations is virtually impossible to predict, and that uncontrolled polymorphic conversions can have a severe impact on the development of molecular materials for potential APIs and speciality chemicals. Bearing in mind the advances made in understanding *some* of the vagaries associated with the solid state, it is sometimes difficult to comprehend why and how new polymorphs still emerge (while others disappear) long after crystal-form screens presumably have been completed. The point is that it can never be stated with certainty that the most stable form has been found; at best it can be determined which of the known forms is the most stable. As the evidence above clearly shows, a new (and most often more-stable) form can appear at any stage in the history of a compound (or life-cycle of a drug).

Today we have access to highly sensitive analytical instruments and automated polymorph screening platforms. High-throughput salt and polymorph screens were “not on the radar” 30–40 years ago, and the whole ethos surrounding drug development was entirely different. In the experiences of one of the authors of this review (R.W.L.) polymorph screens were actually carried out in the past by default, but were not designated as such. During the course of research and development of an API, a large team of highly skilled chemists would fine-tune a process from an initial milligram scale synthesis through scale-up, on to the pilot plant, and ultimately into production. Within reason, time and resources were not the issue. In terms of the pharmaceutical industry, many drugs currently on the market and still highly profitable were developed and produced in this “classic” way.

What has changed? The key differences affecting product development these days are the compressed time scales for drug development (surely at odds with the notion of kinetics and crystallization?) and far fewer skilled process chemists available to develop APIs in the manner described above. The onus today is on efficiency and taking advantage of technical developments that have appeared in the last decade or so. Advances have been made based on the chemistry of the particular compound in question, not least in the development of automated polymorph screening platforms themselves, but also in the increasing sensitivity and precision of automated analytical instrumentation, and in situ analysis and algorithms for pattern recognition (e.g. comparisons of diffraction patterns or Raman spectra).

There is no standard strategy or foolproof recipe for the search for crystal forms. A combination of carefully designed manually performed crystallization experiments combined with automated high-throughput screens can reduce, but not totally eliminate the likelihood of unexpected polymorphic transformations if both highly pure and impure materials are used. All readily accessible “significant” byproducts obtained in the synthesis of a target compound should be considered for use as seeds and additives. There is a need to screen for possible polymorphic transformations under stressed conditions (e.g. extreme humidity, temperature, and pressure) to nurture confidence in the robustness of a product once it leaves the realm of a controlled laboratory space. Furthermore, the screens and solid-state studies should be considered throughout the lifetime of a drug or speciality chemical to allow for the potential materialization of solid phases that take considerable amounts of time to nucleate.

CSP^67^, ^105^, ^106^ methods are now becoming progressively more capable of guiding the search for new crystal forms,^66^, ^121^–^127^ and should be considered in solid-form screening processes, along with knowledge-based hydrogen-bond propensity calculations,^128^–^131^ whenever possible. Since CSP was recently also successfully utilized to determine the crystal structure of a sub-micrometer-sized crystallite (present in picogram amounts) in a bulk consisting of a different polymorph,^132^ it is viable that CSP could aid the structural characterizations of small molecular impurities that potentially act as crystal seeds for the formation of unanticipated crystal forms.

Processes associated with drug and speciality chemical development have changed radically in the last decade or two. Skills associated with particle engineering, drug design, and even relatively “unfashionable” skills such as those concerned with filtration and drying have advanced dramatically. All of these process modifications involve perturbations that can potentially lead to new solid phases.

Whilst advances in technologies associated with form screening, analytical instrumentation, and in silico approaches have come a long way, we are often forced to return to the question of the level of better understanding of the fundamentals associated with crystal nucleation and growth. Will some (or many) of us still “get surprised” by the vagaries of polymorphism and crystallization in 20 years’ time—or will it be all sorted out by then? There are still many challenges and surprises in store.

## Addendum

In the final section of this Review, we will present excerpts from legal proceedings associated with the paroxetine hydrochloride case, with the aim of highlighting the complex relationship between science and the law. Although no scientific aspects of polymorphism are formally discussed, we describe some additional important aspects of the disappearing polymorph phenomenon.

As previously described, the Paxil*®* (paroxetine hydrochloride) litigation (SmithKline Beecham versus Apotex) continued for nearly eight years before the US Supreme Court refused to hear the case. The issues of seeding and inherency, both intimately connected with disappearing polymorphs were central to the case. In a number of instances, the positions of the witnesses (as on record from trial testimony) or of the judge (from his opinion in the first instance) were not always precisely quoted or correctly interpreted in secondary publications. In the interest of informing the reader of how these issues can be interpreted and misinterpreted as well as of setting the record straight, we present some of that testimony and the way it was subsequently interpreted. Please note: one of us (J.B.) was a witness at trial on behalf of SmithKline Beecham.

It will be recalled that Judge Posner found valid the patent for which the independent Claim 1 is simply “Crystalline paroxetine hydrochloride hemihydrate”. However, he also found that Apotex would not be infringing that patent because they would not be marketing an anhydrate API with “high double-digit” percentages of the hemihydrate and would be gaining nothing from the quantities of the hemihydrate that he found would be in the Apotex product. In what follows we relate the events subsequent to Judge Posner’s ruling and how that ruling and the decisions from the Federal Circuit (the venue for patent appeals) were interpreted by the trade press and perhaps some of the community not familiar with the details.

With regard to seeding, there was undisputed evidence that Branford Chemicals (the Apotex subsidiary that actually manufactured the API) started their research on the compound with the hemihydrate, so that their facility was seeded with **Form 1**. Judge Posner found that there would be conversion of anhydrate into hemihydrate:

“*Some conversion from anhydrate to hemihydrate is likely to occur in a seeded facility in which the anhydrate is exposed to air; BCI’s plant is seeded; and the anhydrate manufactured there is exposed to non-dehumidified air before it leaves the plant. The evidence is sufficient to support an inference that BCI will be making at least tiny amounts of the hemihydrate if it is permitted to manufacture the anhydrate*.”

And Judge Posner then related to the amount of hemihydrate and the ability to detect it:

“*In sum, I am not persuaded that Apotex will produce an anhydrate that has sufficient hemihydrate to be detectable by the methods in use in 1985.*”

1985 was the date of the application for the patent on the hemihydrate, but it is unclear why he limited his analysis to 1985, as patent law does not limit the method of detection for infringement purposes to those available at the time of the invention, especially in this case where the claim simply recites “Crystalline paroxetine hydrochloride hemihydrate”. As discussed above, the Federal Circuit ultimately reversed Judge Posner on his non-infringement determination, but ultimately held the claim invalid as inherently anticipated.

In the course of the trial, Judge Posner asked counsel for SmithKline Beecham whether a single crystal of the hemihydrate in anhydrate API would infringe the patent. The response was that in principle yes (in accord with the formal reading of the patent law), but that the SmithKline Beecham case was built on a considerable body of evidence that many of Apotex’s batches had converted and that, therefore, it was highly likely that subsequent batches would exhibit conversion—to an extent much greater than a single crystal and proven as detectable by methods available in 2003.

On appeal, the Federal Circuit agreed with SmithKline Beecham’s claim construction:

“*[N]othing in the ‘723 patent limits that structural compound to its commercial embodiments*,”

and thus overturned Judge Posner on that issue. Moreover, the Federal Circuit found that Apotex would be infringing the **Form 1** patent:

“*Thus, reading Claim 1 in the context of the intrinsic evidence, the conclusion is inescapable that the claim encompasses, without limitation, PHC [Paroxetine Hydrochloride] hemihydrate—a crystal form of paroxetine hydrochloride that contains one molecule of bound water for every two molecules of paroxetine hydrochloride in the crystal structure*.”

In other words, in principle, even a single crystal would infringe the patent. Thus, the Federal Circuit agreed with SmithKline Beecham that Apotex would infringe the patent.

Upon appeal to the Federal Circuit, two of Apotex’s arguments are relevant in the current context. First Apotex attacked the decision acknowledging conversion as clearly erroneous, and, second regarding seeding, they argued:

“*In sum, the district court’s apparent fascination with the seeding theory led it to a finding that smacks of alchemy, not chemistry.*”

At the time of the trial there was still a major misconception about the meaning of a “disappearing polymorph”, as evidenced by the courtroom exchange:

**Question:** “*Okay. And, Dr. Bernstein, under your theory that once hemihydrate was made, seeds of the hemihydrate would contaminate any further paroxetine hydrochloride that was made, then anyone practicing the ‘196 Ferrosan patent in the United States would produce the hemihydrate after the HP23 and 24 batches [SmithKline Beecham’s first batches of **Form 1**] were sent to the United States, correct?*”

**Answer:** “*No that’s not correct. That doesn’t represent my point of view. As you pointed out earlier, the last sentence in my paper says once you have prepared it, you ought to be able to prepare it again. So, I am not saying that the anhydrate can’t be prepared again. What I have said is that after the anhydrate is prepared, if there are—if there are seeds around and water, then it is highly likely that that will convert, and I think you have to make a clear distinction between the preparation and the conversion process. Those are two different processes. I never said it couldn’t be prepared.*”

In April 2004, the Federal Circuit reversed Judge Posner’s ruling and found that *“any amount of crystalline paroxetine hydrochloride hemihydrate without further limitation……will infringe Claim 1.”* However, it found Claim 1 was invalid under a legal argument that the clinical trials that SmithKline Beecham had carried out constituted a “public use.”

The concurring appellate judge in that decision also expressed the view that Claim 1 was invalid because one crystal form converted into a more stable crystal form without human intervention, and was therefore a “naturally occurring process”. However, the two other judges dismissed this view because the crystal compound was a synthetic, man-made compound, and thus is a *“composition of matter”* that is eligible for patent protection.

This decision was appealed to the full court en banc (fifteen judges) in June 2004. The full Court reversed the “public use” issue and remanded the case back to the original panel. In April 2005, the panel again found the claim invalid, but this time on the grounds of inherent anticipation.

“*Because the record contains clear and convincing evidence that the production of PHC anhydrate in accordance with the ‘196 [original Ferrosan anhydrate] patent inherently results in at least trace amounts of PHC hemihydrate, this court holds that the ‘196 patent inherently anticipates Claim 1 of the ‘723 patent……*”

It will be recalled that in US legal terms *inherency* means *invariably* or *inevitably*. Hence, to reach such a conclusion the Federal Circuit would have to be convinced that **Form 1** existed before the SmithKline Beecham inventors said they first detected it late in 1984. To examine the evidence for “*at least trace amounts of PHC hemihydrate*” it is necessary to return to the trial transcript and Judge Posner’s examination of experts from both sides on this question.

The following is the related transcript excerpt of the dialogue between Judge Posner and SmithKline Beecham’s expert:

**Question:** “*I have a few questions, Dr. Bernstein. You said on Friday that there was no hemihydrate before December, 1984. In fact, you said today that you are absolutely convinced there was none. I don’t understand that. I take it you mean there was no detectable hemihydrate, don’t you?”*

**Answer:** “*Well, your Honor, from the history subsequent to December ‘84, when there were locations in which there was definitely hemihydrate and there was water available, there was almost, there was a high, very high probability of conversion. So the fact that there was never any evidence of the hemihydrate prior to 1984 and no evidence of conversion prior to December of 1984 indicates to me that it didn’t exist, and it’s a situation similar to what I described in the case in my own laboratory [in the 1995 review]*.”

In fact, unchallenged data on accelerated stability tests of the anhydrate at 40 °C and 75 % relative humidity over a period of several months in 1982 had exhibited no evidence of any conversion into hemihydrate. Even Judge Posner summarized in his decision:

“*First, a batch of anhydrate manufactured by Ferrosan in 1980, though stored in a hot and humid place (the greater the heat—short of the melting point, of course—and the humidity, the likelier is conversion from the anhydrous to the hemihydrous form), had three years later still not converted to the hemihydrate form, suggesting that it had not been seeded and hence that there were no seeds as late as 1980. And [pilot plant batch] HP22, manufactured just weeks before HP23, contained no detectable hemihydrate, whereas HP23 was entirely hemihydrate.*”

Judge Posner summarized the testimony of Terry Threlfall, Apotex’s witness on this issue:

“*Dr. Terence Threlfall, Apotex’s expert on polymorphism, testified to the contrary of Bernstein that anhydrous and hemihydrous forms of paroxetine can coexist happily. There is support for this conjecture in SmithKline Beecham’s own evidence, of which more later, that some of Apotex’s anhydrous product contains small amounts of hemihydrate without conversion of the rest. In other words, as Threlfall testified, a mixture of anhydrate and hemihydrate can be an equilibrium, in which event the earliest batches of paroxetine manufactured by Ferrosan may have contained undetectable quantities of the hemihydrate. In light of this evidence, Dr. Bernstein’s absolute certainty that hemihydrate did not exist before December 1984 is not tenable. No one knows when the hemihydrate form of paroxetine came into existence, although it is a reasonable inference that it did not exist in a detectable amount until then.*”

The opinion that the hemihydrate did not exist before December 1984 may be untenable in Judge Posner’s view, but there were absolutely no data or scientific evidence that it ever existed before. Moreover, in light of the later observations about the tendency for conversion and the total lack thereof in the 1980 batch strengthened that conviction. With regard to the existence or non-existence of crystal forms, it seems incontrovertible and contrary to the norms of scientific reasoning that one can not claim to have a crystal form for which there is—and up to a certain date has never been—absolutely no physical or chemical evidence for its existence. Especially in light of the results of the accelerated stability tests, the fact that after the date of its appearance some batches did not convert cannot serve as evidence that it existed prior to its actual positive discovery late in 1984.

Furthermore, Judge Posner was discussing the situation after December 1984 in which it was likely that there were seeds of hemihydrate in locations where it have been prepared. The lack of conversion, even in the presence of seeds does not prove his point for the period prior to December 1984. Conversion depended on the amount of water (or humidity), temperature, and pressure. If the combination of those factors was not sufficient, conversion would not take place. In the 1980 accelerated stability tests on a number of samples, they almost certainly would have been sufficient.

This Addendum and some of the legal examples cited in the text provide evidence for the complex relationship—“uneasy bedfellows” in one view—between science and the law, more specifically between the scientific method and scientific reasoning on the one hand and legal reasoning on the other hand. This dynamic relationship has been addressed in a number of monographs^133^–^138^ and will no doubt continue to generate debate from practitioners and scholars of jurisprudence and scientists.

For the scientific community, the standards have been well stated by Peter Huber in the closing paragraph of one of his treatises on the subject:

“*The best test we have of certainty is good science—the science of publication, replication, and verification, the science of consensus and peer review; the science of Newton, Galileo, and Gauss, Einstein, Feynman, Pasteur and Sabin; the science that has eradicated smallpox, polio, and tuberculosis; the science that has created antibiotics and vaccines. Or it is at least, the best test of certainly so far devised by the mind of man.*”^133^

## Biographical Information

*Krešo Bučar obtained a BSc in chemistry with Dr. Ernest Meštrović at the University of Zagreb, and a PhD in chemistry from the University of Iowa with Prof. Leonard R. MacGillivray. He then started his independent research career as a Royal Society Newton International Fellow at the University of Cambridge with Prof. William Jones. While in Cambridge, he was also a Bye-Fellow at Sidney Sussex College. He recently joined the Department of Chemistry at University College London as UCL Excellence Fellow. His research interests mainly concern molecular cocrystals and their applications.*

## Biographical Information

*Robert Lancaster joined Glaxo in 1969, studied part-time to obtain a Grad RIC degree. He gained exposure to the phenomenon of polymorphism in the mid-70s and completed his PhD at the University of East Anglia in 1986. He worked closely with process and pharmaceutical development scientists, specifically looking at issues surrounding all aspects of crystallization and polymorphism. He had some exposure to legal aspects of two high profile Glaxo (GSK) drugs. After retiring from GSK, he joined Prof. Sally Price’s group at University College London on a part-time basis.*

## Biographical Information

*Joel Bernstein studied chemistry at Cornell University (BA 1962) and Yale University (PhD 1967). He was then a postdoctoral fellow with Ken Trueblood at UCLA and then with Gerhardt Schmidt at the Weizmann Institute. He is professor emeritus at Ben-Gurion University of the Negev in Israel, which he joined in 1971 and from which he retired in 2010 as the Barry and Carol Kaye Professor of Applied Science. He is currently Global Distinguished Professor of Chemistry at New York University having taught at Abu Dhabi and Shanghai. His research interests concern chemistry of the organic solid state, particularly polymorphism.*
